# A lipid nanoparticle-based peptide-proteolysis-targeting chimera degrades BCL6 for diffuse large B-cell lymphoma treatment

**DOI:** 10.1007/s12672-026-05153-3

**Published:** 2026-05-03

**Authors:** Shiwei Liu, Xueying Gao, Xuan Ye, Yi Chen, Kai Wang, Yangsui Liu, Sanmei Wang, Haorui Shen, Hongyu Shen, Ran Li, Xiaoyan Qu, Lei Fan

**Affiliations:** 1 Department of Hematology, Jiangsu Province Hospital, The First Affiliated Hospital of Nanjing Medical University, Nanjing, 210029 Jiangsu China; 2 Jiangsu Province Engineering Research Center of Cell and Gene Therapy for Hematologic and Lymphoid Diseases, Nanjing, 210029 Jiangsu China; 3 Center of Hepatobiliary Pancreatic Disease, Xuzhou Central Hospital, Xuzhou, 221009 Jiangsu China

**Keywords:** BCL6, Diffuse large B-cell lymphoma, Peptide, Proteolysis-targeting chimera, Lipid nanoparticle

## Abstract

B-cell lymphoma 6 (BCL6) is an attractive drug target for diffuse large B-cell lymphoma (DLBCL). This study aimed to create a lipid nanoparticle (LNP)-based peptide-proteolysis-targeting chimera (PROTAC), specifically BCL6-PROTAC, to develop effective strategies for treating DLBCL via targeted degradation of the BCL6 protein. Molecular docking, SPR and cellular thermal shift assays revealed that F1324 (Ac-LWYTDIRMSWRVP-OH) is a high-affinity BCL6-binding peptide. PROTAC LNPs were synthesized by modifying F1324 and pomalidomide aptamers onto LNPs via a covalent chemical reaction in a certain proportion. The LNPs were characterized using dynamic light scattering and transmission electron microscopy. The ligand ratio (F1324:pomalidomide) used to verify the optimal BCL6 degradation was 1:5, as determined using western blotting. In vitro and in vivo studies revealed that BCL6-PROTAC significantly inhibited the proliferation of DLBCL cells. Target-specific uptake was used to evaluate the accumulation of BCL6-PROTAC in vivo. Toxicity in normal tissues was detected using H&E staining and serum indices. Overall, we developed a PROTAC that exhibited persistent and excellent BCL6 degradation ability in DLBCL, with an excellent safety profile. Thus, our BCL6 degrader provides a complementary approach to existing clinical‑stage candidates.

**Graphical Abstract:**

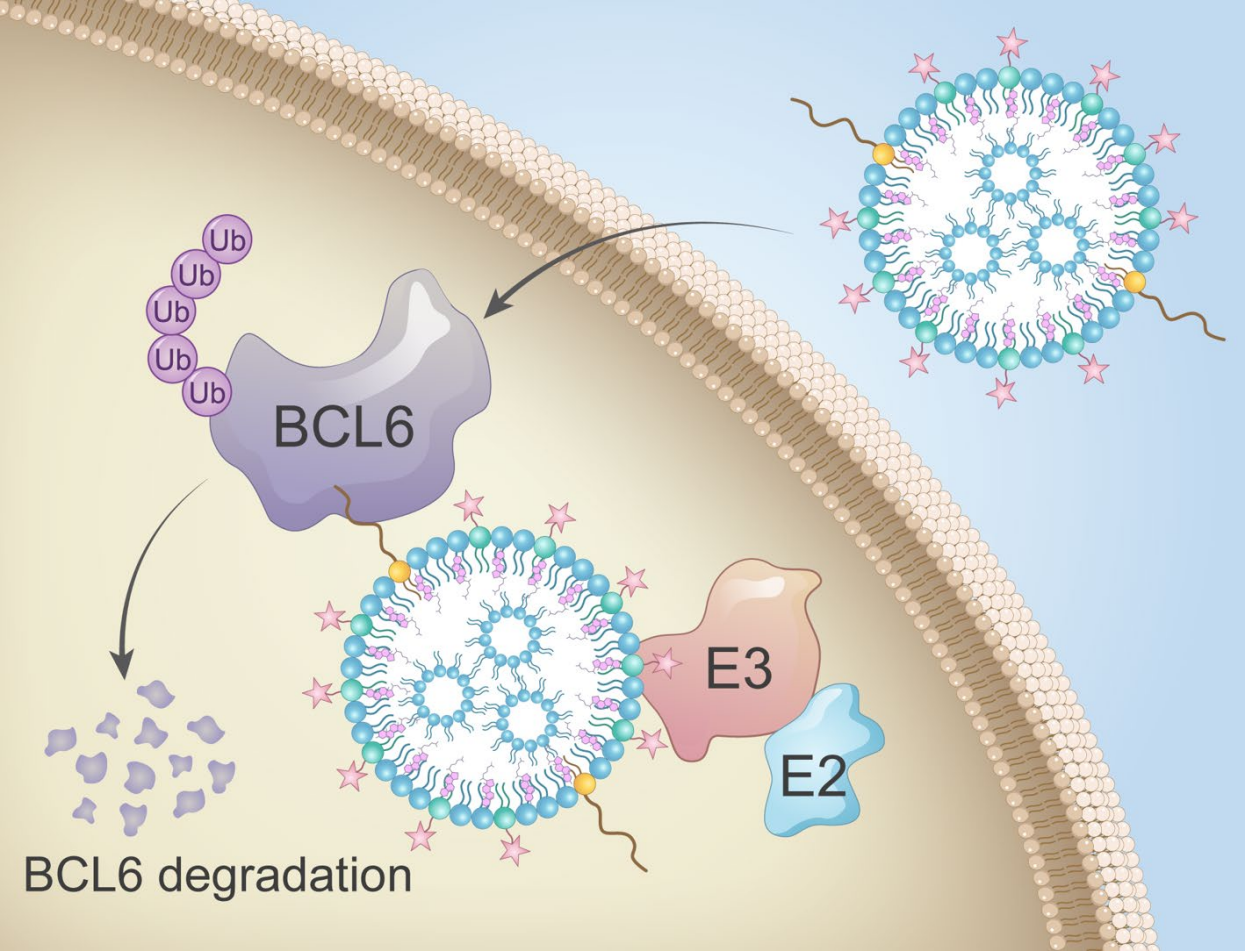

## Introduction

B-cell lymphoma 6 (BCL6) has received increasing attention as a key oncogenic and therapeutic target. BCL6 was first discovered in diffuse large B-cell lymphoma (DLBCL), where its expression is sustained due to chromosomal translocation, somatic hypermutation, and post-transcriptional mechanisms [1–4]. The pro-survival and proliferative functions of BCL6 are necessary for the development and maintenance of germinal centers (GCs) during the humoral immune response; however, when constitutively expressed, GC B cells are susceptible to malignant phenotypes [5, 6]. Many B-cell lymphomas, including the GCB subtype of DLBCL, originate from GC B cells and often depend on aberrant BCL6 expression to maintain their malignant phenotype. Knocking down BCL6 leads to DLBCL cell death in vitro [7]. Constitutive BCL6 expression in vivo increases the risk of DLBCL progression [8, 9]. BCL6 plays an oncogenic role in other hematological malignancies [10]. For example, BCL6 improves the survival and self-renewal rates of primary acute myeloid leukemia cells [11]. Additionally, it contributes to the initiation of chronic myeloid leukemia [12]. Thus, BCL6 is a potential therapeutic target.

Over the past few decades, substantial progress has been made in cancer targeted therapies, with many targeted anti-cancer small molecule drugs approved for cancer treatment. These anti-cancer drugs contain inhibitors and degraders, both of which can effectively reduce the functional activity of target proteins [13, 14]. Despite the promise of small-molecule inhibitors, this treatment has one major drawback: 85% of the proteome lacks suitable binding pockets and cannot be “druggable” [15]. The targeted protein degradation strategy overcomes this limitation of traditional small-molecule inhibitors. Proteolysis-targeting chimera (PROTAC) is a representative targeted protein-degradation technique [16]. PROTAC compounds were developed by combining two molecules that typically do not form complexes together. PROTAC molecules, which contain ligands for the protein of interest (POI) and E3 ubiquitin ligase, can physically bring the POI closer to E3, allowing it to undergo proteasome-mediated degradation [17]. PROTAC reduces the abundance of the protein by binding to any site of the target protein rather than specific sites [18]. Therefore, it is a substantially promising therapeutic strategy. To date, many PROTACs that target pathogenic proteins have been successfully developed. Recently, a research team identified NR4A1 as a target for cancer immunotherapy and developed the first PROTAC targeting NR4A1, named NR-V04. NR-V04-mediated NR4A1 degradation is a new approach for tumor therapy [19]. Similarly, knocking out HPK1 using PROTAC is considered an effective way to enhance the anti-tumor immune response [20]. Additionally, MS8847, a novel EZH2 PROTAC degrader, exerts anti-proliferative effects on tumor cells [21]. Currently, most PROTACs use small-molecule inhibitors to target warheads for POI; therefore, many proteins with warhead scarcity are considered “undruggable” [22]. With the rapid development of structural biology and high-throughput virtual screening, it is becoming increasingly convenient to obtain peptides with a high affinity for POI that are not amenable with small-molecule inhibition [23]. Therefore, the synthesis strategy of PROTAC using peptides has circumvented the constraints encountered by small-molecule PROTACs, which necessitate shallow binding pockets, thereby expanding the scope of “undruggable” protein degradation [24]. Many peptide-PROTACs (p-PROTACs) have been designed and developed. FOXM1-targeting peptides were screened from a phage display library and a p-PROTAC that can degrade FOXM1 was synthesized [25]. Our previous study suggested that p-PROTAC, which targets the degradation of PTGES3, is a promising drug for the treatment of hepatocellular carcinoma [26]. Recently, a p-PROTAC drug that specifically targets BRCA2 was developed, and combination therapy with PARP inhibitors promoted cell death in prostate cancer cells [27]. Additionally, compared with traditional small-molecule-based PROTAC, p-PROTAC has high specificity and low toxicity [28]. Therefore, the design and synthesis of p-PROTACs capable of degrading core oncogenic proteins may provide a useful starting point for further investigation into anti-DLBCL strategies and related mechanistic studies.

In this study, we proposed an engineered p-PROTAC template capable of linking peptides and pomalidomide through lipid nanoparticles (LNP) for the degradation of the endogenous BCL6 protein in DLBCL cells. To accomplish this, we first validated the binding of the previously reported F1324 peptide to BCL6. Subsequently, the compounds were synthesized and characterized. We characterized dose- and time-dependent degradation of p-PROTAC and obtained an optimal formulation. Finally, we showed that BCL6-targeting p-PROTACs inhibited the proliferation of DLBCL cells in vitro and in vivo. Collectively, this study expands the perspective of researchers on DLBCL treatment and shows that p-PROTAC has the potential to become an effective drug in the future.

## Materials and methods

### Molecular docking

The PDBePISA online server (https://www.ebi.ac.uk/pdbe/pisa/) was used to identify the possible binding sites between BCL6 protein and F1324 peptide. Table 1 shows the interface results. Δ^i^G means the solvation free energy gain upon formation of the interface, negative value represents hydrophobic interfaces. Δ^i^G *P*-value shows the *P*-value of the observed solvation free energy gain. If *P* < 0.5, it indicates that the hydrophobicity of the interfaces is significant, which means that the interface surface may be interaction-specific.

**Table 1:** PDBePISA interface result of F1324 and BCL6

Structure 1	Structure 2	Interface area (A^2^)	Δ^i^G (kcal/mol)	Δ^i^G (*P*-value)
F1324	BCL6	425.4	−7.8	0.405

### Synthesis of F1324 peptide

The peptide synthesis was entrusted to ChomiX Biotech Company (Nanjing, China). The F1324 peptide was synthesized by the standard solid-phase peptide synthesis method. The synthesis was performed on a Rink amide MBHA resin as a solid phase carrier and each 9-fluorenylmethyloxycarbonyl-protected amino acid was coupled sequentially. The peptide was purified by high-performance liquid chromatography with a purity > 95%. Finally, electrospray ionization mass spectrometry was used to confirm the molecular weight matched the theoretical value.

### Surface plasmon resonance (SPR) analysis

The interaction between BCL6 protein and F1324 peptide was analyzed by SPR using a Biacore T200 system. BCL6 was immobilized on a CM5 chip by amine coupling. Various concentrations of F1324 (8, 4, 2, 1, 0.5, 0.25 and 0 µM) were injected over the surface, and binding responses were monitored in real time. Kinetic constants were determined by fitting the sensorgrams to a 1:1 binding model.

### Cell lines and culturing

Human DLBCL cell line SU-DHL-4 was obtained from Sunncell Biotechnology Co., Ltd (Wuhan, China). Human DLBCL cell line OCI-Ly1 was stored in our laboratory. The suppliers provided cell line authentication. The above cells were cultured in 1640 medium supplemented with 10% FBS, along with 1% penicillin and 1% streptomycin, and incubated at 37℃ in a cell culture incubator with 5% CO_2_. The cell lines were stored in liquid nitrogen for subsequent use.

### Western blotting analysis

Firstly, the cells were obtained by centrifugation at 800 g for 15 min at 18–25℃. The harvested cells were washed three times with PBS. The total protein lysate from indicated cells was obtained by Universal Total Protein Extraction Kit with Strong Lysis Buffer and Spin Column (P0013WM, Beyotime Biotechnology) per the instructions. The determination of protein concentration in lysate depends on BCA Protein Detection Kit (P0012S, Beyotime Biotechnology). 5 × SDS-PAGE Sample Loading Buffer (P0015L, Beyotime Biotechnology) was added to the lysate and the mixture was heated at 95℃ for 10 min. Subsequently, equal amounts of proteins from each sample underwent SDS-PAGE electrophoresis and immunoblotting. The membrane was sequentially detected by the primary antibody and incubated with the second antibody, and finally exposed through a chemiluminescence reaction system (SCG-W3000 PLUS, Servicebio Technology Co., Ltd). Images were quantified using Image-Pro Plus 6.0 software. The first antibodies used in this study were anti-BCL6 (ab33901, abcam), anti-PCNA (10205-2-AP, Proteintech Group) and anti-β-actin (66009-1-Ig, Proteintech Group). The second antibodies were purchased from the Proteintech Group (SA00001-1 and SA00001-2).

### BCL6 degrader LNPs production

LNPs were formulated by microfluidic mixing using a previously described method [29]. Briefly, ethanol solutions containing DSPC, cholesterol, DSPE-mPEG2k, DSPE-PEG2k-pomalidomide and DSPE-PEG2k-peptide, at molar ratios of 48:38:11:1.5:1.5 (1:1), were mixed with 10 mM PBS buffer using a microfluidic mixer at a ratio of 1:3. Prepared solutions were mixed via a microfluidic mixing device at a flow rate of 12 ml/min to obtain LNPs. Then, prepared LNPs were dialyzed against PBS using 100k Molecular Weight Cutoff dialysis cassettes (Merck Millipore) overnight. Similarly, the BCL6 degrader LNPs (1:5) and (5:1) were successfully synthesized as per the aforementioned method. Size distribution and PDI of LNPs were determined via dynamic light scattering using a Zetasizer Nano ZSP (Malvern Instruments, UK).

### Quantitative reverse transcription-polymerase chain reaction (qRT-PCR)

First, total RNA was isolated from DLBCL cells using an RNApure Tissue&Cell Kit (CW0584, CWBIO). Next, Complementary DNA generation was conducted using an HiFiScript first strand cDNA synthesis Kit (CW2569, CWBIO) according to the manufacturer’s protocols. Finally, real-time PCR was performed using UltraSYBR Mixture (CW0957, CWBIO) on an Applied Biosystems Real-Time PCR System. All measurements were taken three times and normalized to endogenous levels of GAPDH. The specific qRT-PCR primer sequences were listed below: BCL6-forward, 5-GGAGTCGAGACATCTTGACTGA-3, BCL6-reverse, 5-ATGAGGACCGTTTTATGGGCT-3; GAPDH-forward, 5-GACACCCACTCCTCCACCTTT-3, GAPDH-reverse, 5-TCTCTTCCTCTTGTGCTCTTGCT-3.

### Cell proliferation assay

Cell counting kit-8 (CCK-8) assay was used to detect the proliferation ability of DLBCL cells. Cells were resuspended in culture medium and inoculated in 96-well plates at an appropriate density of 5000 cells per well. After treatment of F1324/BCL6-PROTAC, cell samples were incubated (37℃, 2 h) with CCK-8 solution (C0037, Beyotime Biotechnology). Following incubation, the absorbance of each well at 450 nm was measured by spectrometer at the indicated time points. Each experimental group had 3 independent samples.

### Animal studies

To generate a tumor xenograft model, 5 × 10^6^ SU-DHL-4 cells were resuspended in 100 µl 1640 medium without FBS and injected into the subcutaneous tissue of each 5-week-old BALB/c nude mouse (5 BALB/c nude mice per group). One week after planting the cells, nude mice were intraperitoneally injected with normal saline / F1324 / BCL6-PROTACs, every other day for 2 weeks. At 9 weeks of age, BALB/c nude mice were euthanized by CO_2_ and implanted tumors were obtained and weighed. The tumor volumes were measured once a week. The weight of BALB/c nude mice were measured every three days. Xenograft tumors were subjected to western blotting analysis, hematoxylin and eosin (H&E) staining and immunohistochemistry (IHC). To further investigate toxicity, H&E staining on major organs (lung, liver, spleen, kidney and heart) were conducted. Tumor volume was calculated utilizing the following formula: volume (mm^3^) = 0.5 × length × width × width.

For noninvasive fluorescence imaging and tracking, we labeled the BCL6-PROTAC with 1,1’-dioctadecyl-3,3,3’,3’-tetramethylindotricarbocyanine iodide (DiR iodide). The in vivo distribution of DiR iodide-labeled BCL6-PROTAC was visualized by near-infrared fluorescence (NIR) in vivo imaging, with the fluorescence signal intensity (unit: p/sec/cm^2^/sr) directly reflecting the PROTAC accumulation and biodistribution in the tumor-bearing mouse model.

### H&E staining

The tissue samples were fixed by 10% formalin solution and embedded in paraffin. Next, paraffin blocks underwent sectioning at a thickness of 4–6 μm. Subsequently, the tissue sections were deparaffinized using xylene and then rehydrated with graded ethanol. After undergoing hematoxylin staining and eosin counterstaining, slices were sealed with neutral resin and observed under a microscope after 24 h.

### IHC

For the IHC, the tissue sections firstly underwent antigen retrieval to enhance the specificity of antibody binding. 10% normal goat serum was applied to the slices at room temperature for 30 min to block any non-specific binding sites. Subsequently, the slices were incubated with anti-Ki67 (27309-1-AP, Proteintech Group) and corresponding secondary antibody at room temperature, respectively. Immunohistochemical staining showed brownish yellow granular deposition in the nucleus of DLBCL cells.

### Serum liver and kidney indexes in nude mice

The supernatant was obtained by centrifuging blood samples from nude mice. The reagent kits (S03030, S03040, S03036 and S03076, Rayto) were used to measure alanine aminotransferase (ALT), aspartate aminotransferase (AST), blood urea nitrogen (BUN) and creatinine (CREA).

### Statistical analysis

Statistical analysis and graph generation were performed with GraphPad Prism 8.0.1 software. This study involved three independent experiments, with results displayed as mean ± s.d. or a representative photograph. Student’s t-test and ANOVA were applied for data analysis, considering *P < 0.05, **P < 0.01, ***P < 0.001 and ****P < 0.0001 as statistical significance.

## Results

### Identification the high affinity between BCL6 and F1324 peptide

**Fig. 1:**
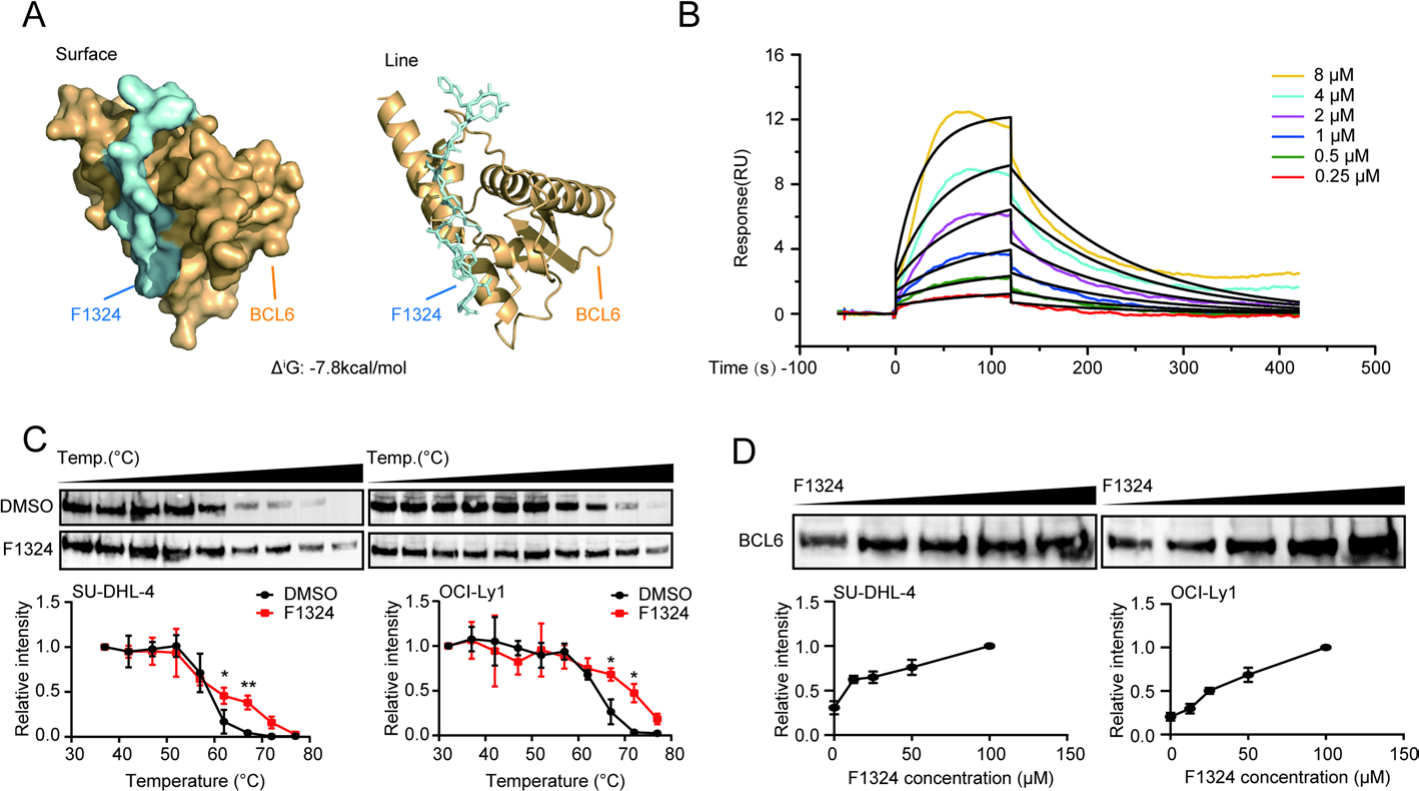
Identification of the high affinity between F1324 peptide and BCL6 protein. **A** The optimized binding modes between F1324 peptide and BCL6 protein, Blue: F1324; Yellow: BCL6 protein (5-129). **B** The combination analysis of F1324 peptide and BCL6 protein by surface plasmon resonance. **C** Temperature-dependent cellular thermal shift assay indicated the high thermal stability of BCL6 in SU-DHL-4 and OCI-Ly1 cell lysates treated with F1324 peptide. **D** Concentration-dependent cellular thermal shift experiment displayed the elevated thermal stability of BCL6 treated with F1324 peptide in SU-DHL-4 and OCI-Ly1 cell lysates at 67℃, respectively

PROTAC is a promising protein degradation technology for drug development that promotes targeted degradation of POI [30, 31]. p-PROTACs use peptides to bind to POI [32]. To successfully synthesize a p-PROTAC that can degrade BCL6, we needed to obtain a high-affinity BCL6-binding peptide. A previous study reported a novel BCL6-binding peptide, F1324 (Ac-LWYTDIRMSWRVP-OH) [33]. To explore the interactions between F1324 and BCL6, PDBePISA was used. The predicted model of F1324 and BCL6 (5-129) illustrated the interactions between these two molecules. Figure 1A shows that F1324 contacts BCL6, with blue indicating F1324, and yellow indicating BCL6. Detailed information regarding the interface contact areas and estimated free energies of the interactions are listed in Table 1. F1324 interacted with BCL6 with an estimated binding Δ^i^G of − 7.8 kcal/mol. The hydrogen bonds predicted to be formed between F1324 and BCL6 (5-129) showed that the main binding site residues included F1324 TRP10 and BCL6 MET51. SPR was used to verify the binding affinity between F1324 and BCL6. Figure 1B shows the stable dynamic binding process between BCL6 and F1324 at different concentrations. The maximum binding response gradually increased with increasing concentration until saturated. After the 120-s bonding phase, the response units decreased with the removal of F1324, indicating that F1324 gradually dissociated from the BCL6 surface. To further demonstrate the binding between molecules, we used a cellular thermal shift assay (CETSA), in which the binding of peptides to BCL6 resulted in increased BCL6 protein stability and higher melt temperatures induced protein unfolding. Both temperature- and dose-dependent CETSA results [34] indicated that F1324 increased the thermal stability of BCL6 in DLBCL cells, illustrating the strong binding of F1324 to BCL6 (Fig. 1C and D). The results of the above experiments provide strong evidence for a direct interaction between F1324 and BCL6, which supports F1324 as a candidate peptide for synthesizing a BCL6-PROTAC.

### BCL6-degradation LNP fabrication and characterization

**Fig. 2:**
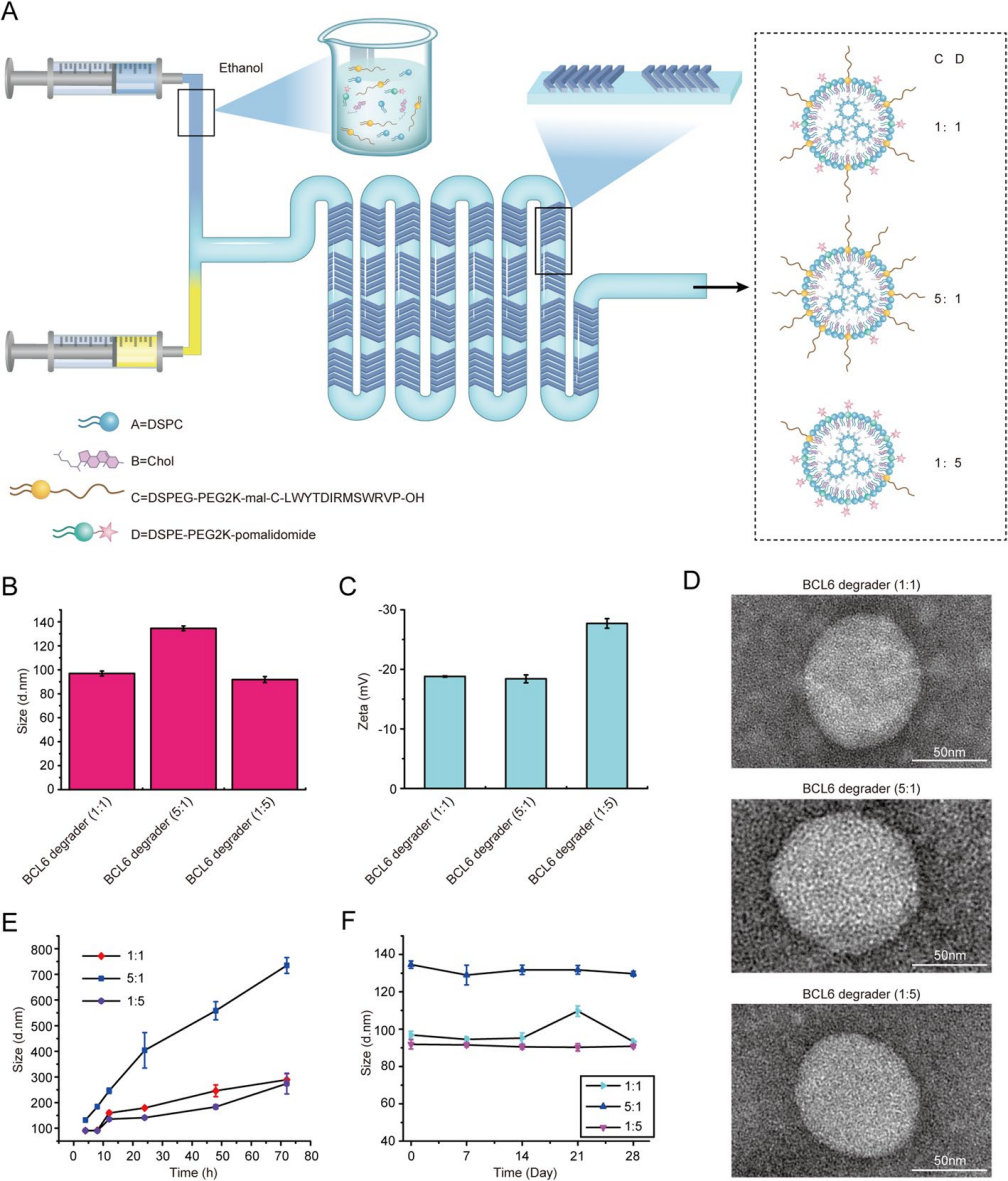
Synthesis and characterization of LNPs. **A** The flowchart of BCL6 degraders synthesis. **B** Particle size distribution of BCL6 degraders detected via dynamic light scattering. **C** Zeta potential of BCL6 degraders. **D** The picture of BCL6 degraders was obtained via transmission electron microscope. **E** The stability of BCL6 degraders in the 5% bovine serum medium was tested. **F** The stability of BCL6 degraders in phosphate buffered saline was tested

Although p-PROTACs successfully degrade several targets, they exhibit membrane impermeability and low stability [35]. Low permeability and poor stability severely limit the deployment of p-PROTACs as a generalized therapeutic modality. Owing to their similar composition to that of the cell membrane, LNPs easily fuse with receptor cells and exhibit high transfection efficiency [36]. Cholesterol, a neutral lipid in LNPs, enhances membrane fusion and transfection efficiency and promotes particle stability [37, 38]. Therefore, LNP represent a marked therapeutic vehicle that has allowed us to develop strategies for improving the potency of p-PROTAC by improving its permeability and stability. Figure 2A depicts BCL6 degrader LNPs (including DSPE-PEG2k-F1324 and DSPE-PEG2k-pomalidomide) synthesis. To optimize the ligand ratios of BCL6 degrader LNPs, we set the F1324 to pomalidomide ratios to 1:1, 5:1, and 1:5. The size of the BCL6 degraders was determined using dynamic light scattering (DLS). Specifically, the mean hydrodynamic sizes of BCL6 degrader (1:1), (5:1), and (1:5) were 96.92, 134.6, and 91.87 nm, respectively (Fig. 2B). The size of BCL6 degrader (5:1) was slightly larger than that of the other two BCL6 degraders. Additionally, the mean zeta potential of BCL6 degrader (1:1), (5:1), and (1:5) measured using DLS was approximately − 18.8, − 18.4, and − 27.7 mV, respectively (Fig. 2C). The change in zeta potential demonstrated the synthesis of BCL6 degraders with different ligand ratios. Transmission electron microscopy (TEM) images indicated that the BCL6 degraders possessed a well-dispersed and uniform spherical morphology (Fig. 2D). We also measured the stability of the BCL6 degraders. In the 5% bovine serum medium at 37 ℃, the particle size of the BCL6 degrader (1:1) and (1:5) groups did not significantly change within 24 h, indicating the excellent colloidal stability of these two LNPs under the in vitro conditions that simulate physiological environments (Fig. 2E). Regarding storage stability, the change in LNP size of the three types of BCL6 degraders in PBS was small after 28 days at -20℃(Fig. 2F). Material characterization revealed that BCL6 degraders with different ligand ratios were successfully synthesized and exhibited good stability in both storage and simulate physiological environments.

### The effect of BCL6 degraders on BCL6 protein degradation

**Fig. 3:**
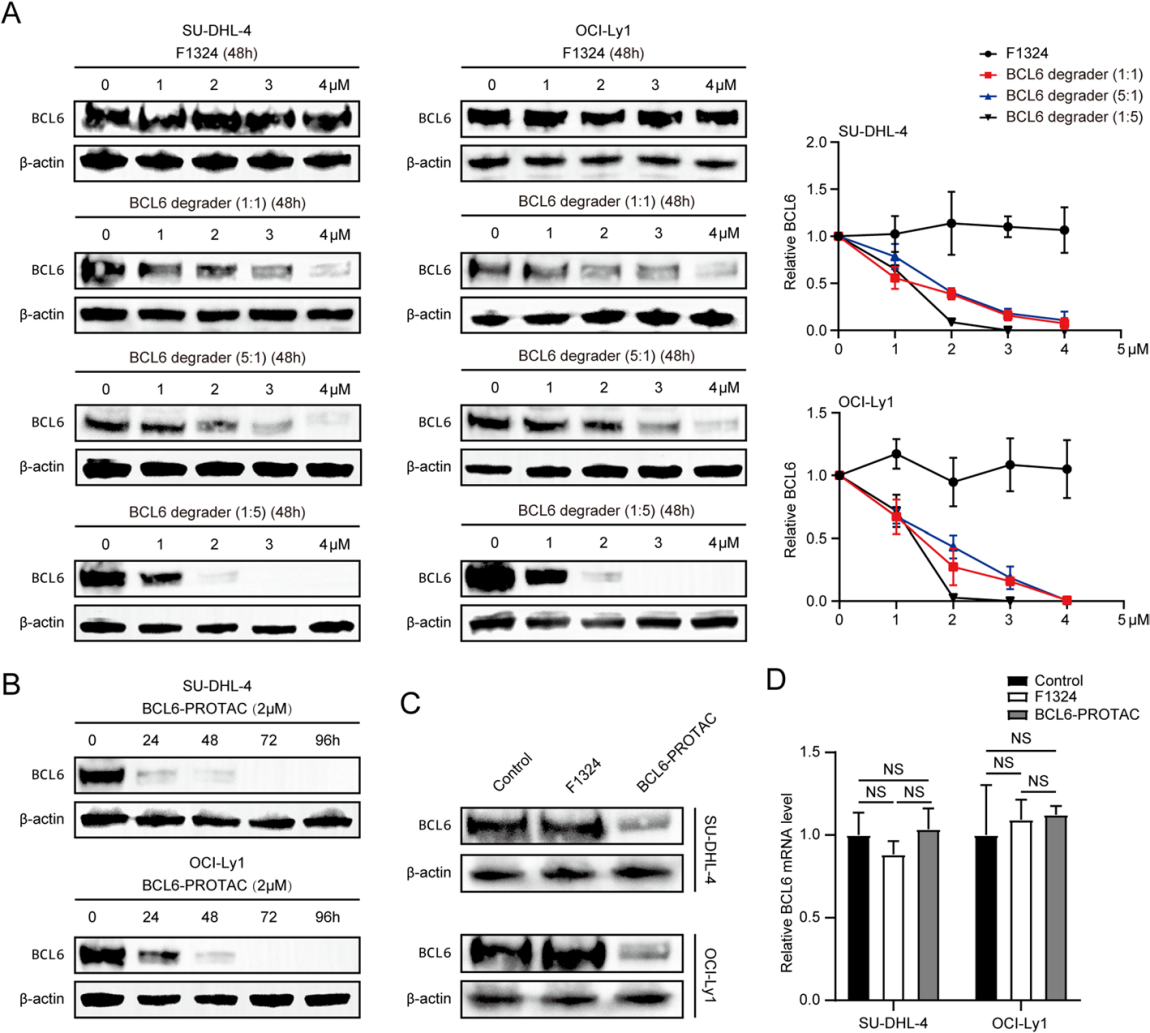
Determination of optimal ligand ratio. **A** The expression level of BCL6 protein in diffuse large B-cell lymphoma (DLBCL) cells treated with different doses of F1324 or BCL6 degraders was detected by western blotting. **B** The expression level of BCL6 protein in DLBCL cells treated with 2 µM BCL6-PROTAC at different time points was detected by western blotting. **C** Western blotting analysis of the protein levels of BCL6 in DLBCL cells treated with F1324 or BCL6-PROTAC. **D** The qRT-PCR analysis of the mRNA levels of BCL6 in DLBCL cells treated with F1324 or BCL6-PROTAC

We evaluated which degrader had the best ability to degrade the BCL6 protein. To verify the effectiveness of the BCL6 degraders in vitro, we performed western blotting to determine the expression of BCL6 in SU-DHL-4 and OCI-Ly1 cells transfected with BCL6 degraders. After 48 h of treatment, the BCL6 degraders induced a dose-dependent decrease in BCL6 protein levels in DLBCL cells and the ratio of 1:5 (F1324:pomalidomide) showed the best BCL6 degradability compared with that of the other ratios (Fig. 3A). Therefore, we established the BCL6 degraders (1:5) as BCL6-PROTACs. Time course studies showed that the BCL6 protein is rapidly and nearly completely depleted within 48 h and remains low up to 96 h, with no obvious rebound (Fig. 3B). To investigate the specific molecular mechanism of PROTAC degradation of the BCL6 protein, we measured the transcription and translation levels of BCL6 in DLBCL cells after BCL6-PROTAC treatment. Western blotting and PCR indicated that BCL6-PROTAC selectively reduced the BCL6 protein level rather than mRNA levels, supporting the conclusion that BCL6-PROTAC acts at the post-translational level (Fig. 3C and D). Collectively, our data revealed that BCL6 degrader (1:5) is an effective BCL6 degrader in DLBCL cells.

### BCL6-PROTAC inhibits DLBCL cell growth in vitro and in vivo

**Fig. 4:**
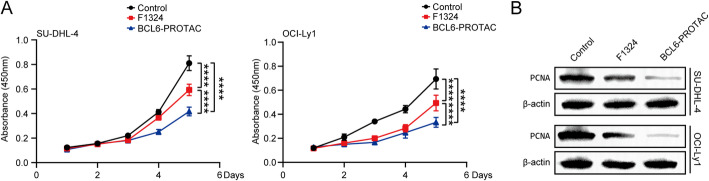
BCL6-PROTAC inhibits proliferation of diffuse large B-cell lymphoma (DLBCL) cells in vitro. **A** CCK-8 assays showed the proliferative ability of DLBCL cells treated with F1324 or BCL6-PROTAC. **B** Western blotting assays showed the expression level of PCNA protein in DLBCL cells treated with F1324 or BCL6-PROTAC

**Fig. 5:**
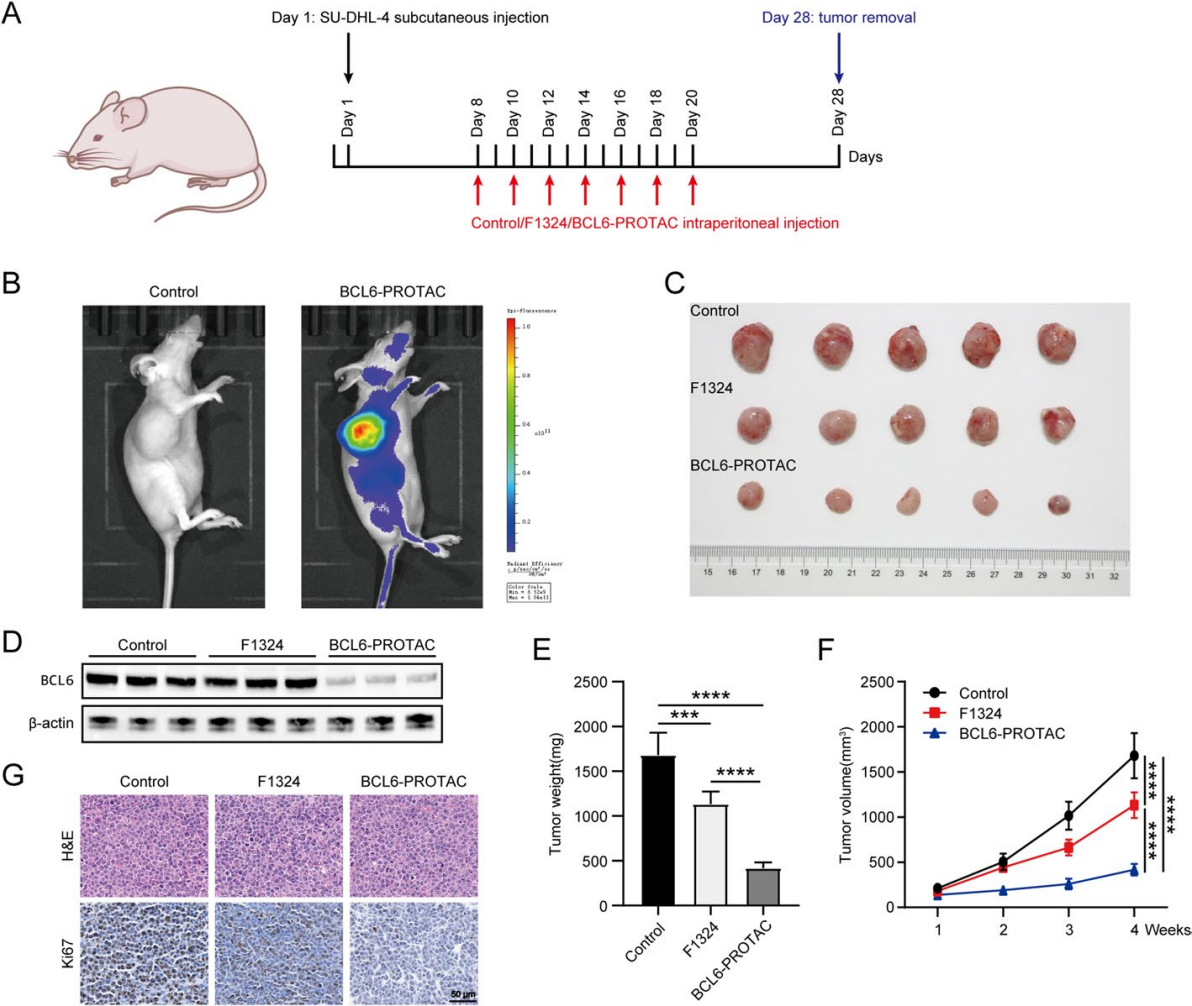
BCL6-PROTAC inhibits proliferation of diffuse large B-cell lymphoma (DLBCL) cells in vivo. **A** The protocol of F1324 or BCL6-PROTAC treatment in vivo. **B** The in vivo distribution of DiR iodide-labeled BCL6-PROTAC. **C** DLBCL tumor picture from xenograft tumor models after 28 days. **D** Western blotting assays showed the expression level of BCL6 protein in DLBCL tumors from xenograft tumor models injected with F1324 or BCL6-PROTAC. **E** Statistical diagram of tumor weight of nude mice injected with DLBCL cells in experimental group and control group. **F** Statistical diagram of tumor volume of nude mice injected with DLBCL cells in experimental group and control group. **G** The immunohistochemistry images of Ki67 protein of xenograft tumors from experimental group and control group

BCL6 is an oncoprotein linked to the regulation of proliferation [39]. Following the demonstration of BCL6 degradation by BCL6-PROTAC, proliferative phenotypic effects were explored using DLBCL cell lines. We conducted a CCK8 assay using SU-DHL-4 and OCI-Ly1 cells to determine whether BCL6-PROTAC-mediated degradation of BCL6 altered the proliferative ability of DLBCL cells. Figure 4A showed that BCL6-PROTAC treatment significantly decreased the proliferation of DLBCL cell proliferation ability. To further validate the proliferation-inhibitory ability of BCL6-PROTAC, western blotting for PCNA was performed on DLBCL cells (Fig. 4B). Compared with the control group, this proliferation marker of these two types of DLBCL cells treated with BCL6-PROTAC were significantly reduced. Having identified the proliferative phenotypic changes in vitro attributable to BCL6-PROTAC-mediated BCL6 degradation, nude mouse transplanted tumor models were established using SU-DHL-4 cells in an in vivo setting. The anti-cancer function of BCL6-PROTAC in vivo was studied after its intraperitoneal injection (Fig. 5A). The enhanced permeability and retention (EPR) effect refers to a universal pathophysiological phenomenon and mechanism in which selective accumulation of nanomedicines in tumor tissues occurs via the hyperpermeable tumor vasculature, resulting in local high drug concentration [40, 41]. Figure 5B shows the delivery distribution and efficiency of BCL6-PROTACs carrying DiR iodides in tumor-bearing nude mice. Notably, this nanomedicine can efficiently aggregate at the tumor site, relying on the EPR effect. To further validate the xenograft model, ex vivo analysis was performed on tumors resected 28 days after DLBCL cell implantation (Fig. 5C). The expression levels of the BCL6 protein identified using western blotting in mouse xenograft lysates confirmed the previous observations (Fig. 5D). Notably, LNP-mediated BCL6 degradation significantly inhibited tumor growth (Fig. 5E and F). Similarly, BCL6-PROTAC treatment significantly decreased the expression of the proliferation marker, Ki67, in tumor tissues (Fig. 5G). Collectively, in vitro and in vivo depletion of BCL6 by BCL6-PROTAC inhibited DLBCL cell growth.

### BCL6-PROTAC exhibits an excellent safety profile in mice

**Fig. 6:**
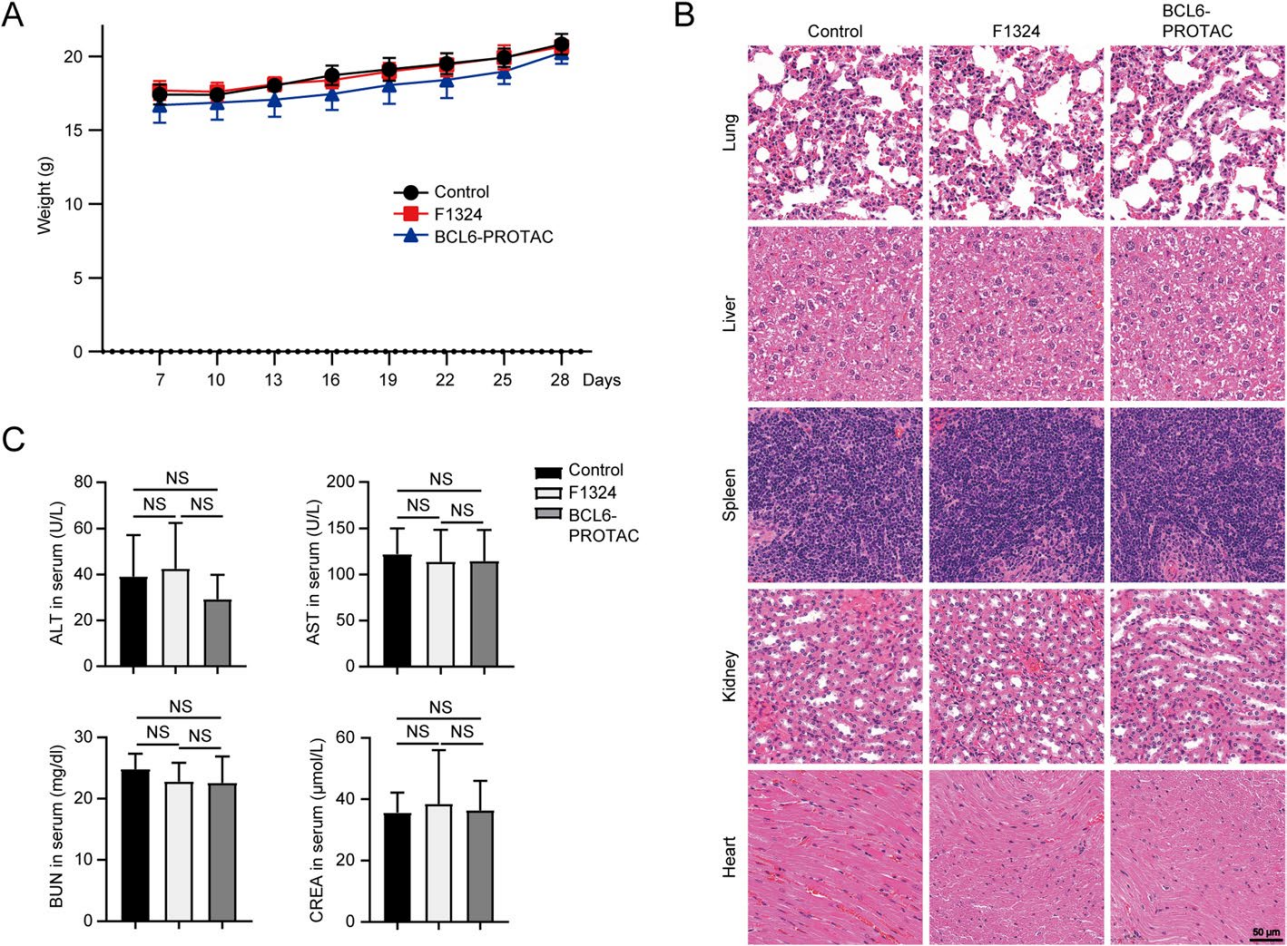
BCL6-PROTAC is non-toxic to major organs. **A** Changes of body weight of tumor-bearing nude mice injected with SU-DHL-4 cells. **B** Hematoxylin and eosin staining of vital organ tissues harvested from xenograft tumor models subcutaneously injected with SU-DHL-4 cells. **C** Activities of serum alanine aminotransferase, aspartic acid transferase, creatinine and blood urea nitrogen from nude mice from experimental group and control group

We evaluated the toxicity of BCL6-PROTAC in BALB/c mice. Figure 6A shows that there was no significant change in the body weight of mice after intraperitoneal injection of BCL6-PROTAC. We performed histological examinations using H&E staining to evaluate the lungs, livers, spleens, kidneys, and hearts of the mice on day 63. Notably, BCL6-PROTAC treatment did not cause any observable damage to vital organs (Fig. 6B). Additionally, the serum hepatic or renal function indices indicated no abnormalities in any group (Fig. 6C). These findings provide important preclinical evidence for the potential clinical application of BCL6-PROTAC as a non-toxic and effective anti-DLBCL agent.

## Discussion

Although many PROTACs for BCL6 degradation have been reported [42–45], their therapeutic effects in clinical applications are unsatisfactory. In this study, we successfully synthesized BCL6-PROTAC, a PROTAC that binds different ligands based on LNPs, and adjusted the ratio of ligands to achieve optimal degradation of BCL6. BCL6-PROTAC significantly inhibited DLBCL cell proliferation in vitro and *in vivo.* Our findings confirm that BCL6 is an effective target for DLBCL treatment.

Compared to traditional inhibitor drugs, PROTACs have a longer-lasting therapeutic effect because they irreversibly eliminate carcinogenic proteins. Although PROTACs have outstanding characteristics in cancer treatment, their clinical translation has stagnated owing to their inherent structural defects [30]. First, the high molecular weight of PROTAC results in a low permeability. Second, the poor water solubility of PROTACs leads to low serum stability and treatment failure. Therefore, poor water solubility and low cell permeability substantially limit their pharmacokinetic properties, which require the use of appropriate delivery systems for improvement [46]. Various carriers have been explored for PROTAC delivery, including organic and inorganic nanoparticles. Nanoparticles have long been used to optimize water solubility and pharmacokinetic properties of drugs. Additionally, nanoparticles can provide longer blood circulation times owing to their high stability [47]. Several nanoparticle-based delivery systems have been approved by FDA due to their ability to regulate the physicochemical properties of drugs [48]. Organic nanoparticles used for drug delivery include polymeric nanoparticles and LNPs. Polymeric nanoparticles can load drugs and their surfaces can also be easily modified to obtain additional functions [49]. The PEG-PLGA copolymer is a popular drug carrier for the development of PROTAC. The blood half-life of the PEG-PLGA copolymer loaded with PROTAC was increased by slowly releasing PROTAC [50]. Liposomes and LNPs can load a wider range of drug types than polymeric or inorganic nanoparticles. Our previously developed PROTAC that used liposome delivery to load peptides and pomalidomides, which successfully degraded the PTGES3 protein [26]. A recent study revealed that the use of liposomes co-encapsulating PROTAC and nintedanib for the treatment of vemurafenib-resistant melanoma achieved satisfactory results [51]. Additionally, galactose-coated liposome delivery has good tumor-targeting abilities, demonstrating improved delivery efficiency [52]. Notably, researchers have used cell-derived biomimetic LNPs extracted from homologous cancer cells to encapsulate PROTAC, achieving high stability and drug compatibility [53]. Inorganic gold nanoparticles (GNPs) have also been reported as drug carriers for the development of PROTAC. GNPs loaded with cetirizine and pomalidomide can successfully degrade the ALK protein and have the potential to serve as an alternative treatment for patients resistant to ALK inhibitors [54]. In this study, we synthesized a highly stable PROTAC using LNPs, which modified peptides and pomalidomides on its surface in certain proportions. This synthetic approach has the potential to be extended to the development of PROTAC for other pathogenic proteins.

Two PROTACs targeting BCL6, BMS-986,458 [55] and ARV-393 (Clinical findings for ARV-393 remain limited to meeting abstracts and posters), have been reported, both of which exhibit strong degradation activity and promising anti‑tumor effects in preclinical DLBCL models. However, these BCL6 degraders have limited flexibility in regulating degradation efficiency. In contrast, our LNP‑formulated BCL6‑PROTAC presents a unique advantage that can be adjusted for degradation efficiency by further optimizing the ligand ratio. This feature high the potential of our BCL6‑PROTAC as a complementary alternative to BMS‑986,458 and ARV‑393 for targeted therapy. Surprisingly, we observed a significant inhibition of cell proliferation in vitro and in vivo by the parent compound F1324. This new LNP based peptide PROTAC not only targets the degradation of BCL6 protein, but its parent compoundF1324 can also act as an inhibitory peptide that suppresses residual BCL6 function. Therefore, BCL6-PROTAC is a powerful system that combines the inhibitory function of F1324 with the protein degradation ability of PROTAC. Furthermore, the biodistribution of the injected BCL6-PROTACs indicated that this PROTAC had a strong specificity and targeting performance using the EPR effect. In the future, based on the characteristics of LNPs loaded with multiple drugs, we will continue to modify PROTAC to exert multiple biological functions.

## Conclusion

We developed an integrated strategy for the degradation of target proteins using LNPs, which was successfully applied to PROTAC synthesis and functional validation. The LNPs, designated BCL6-PROTAC, exhibited good targeting capabilities towards DLBCL tissue, promoting the aggregation of degrader systems at tumor sites. Additionally, the degradation effect was optimized by adjusting the proportion of ligands in this PROTAC. BCL6-PROTACs effectively degraded BCL6 and inhibited DLBCL cell proliferation, exhibiting excellent in vivo anti-tumor activity and low toxicity. Therefore, this study provides a novel PROTAC design scheme, and a better understanding of the relationship between BCL6 and DLBCL. Notably, LNP, as a vehicle, require further investigation of their functional characteristics to determine the anti-cancer efficacy of BCL6-PROTAC. Overall, this proof-of-concept study highlights the superiority of LNP carriers in PROTAC synthesis and accelerates the development of LNP-based anti-tumor drugs.

## Data Availability

The BCL6 macromolecular structural information (PDB ID: 1R29) analysed during the current study are available in the public RCSB PDB database (https://www.rcsb.org/).

## Abbreviations

BCL6: B cell lymphoma 6
DLBCL: Diffuse large B-cell lymphoma
GCs: Germinal centers
PROTAC: Proteolysis-targeting chimera
POI: Protein of interest
p-PROTAC: Peptide-PROTAC
LNP: Lipid nanoparticles
SPR: Surface plasmon resonance
qRT-PCR: Quantitative reverse transcription-polymerase chain reaction
CCK-8: Cell counting kit-8
H&E staining: Hematoxylin and eosin staining
IHC: Immunohistochemistry
ALT: Alanine aminotransferase
AST: Aspartate aminotransferase
BUN: Blood urea nitrogen
CREA: Creatinine
CETSA: Cellular thermal shift assay
DLS: Dynamic light scattering
TEM: Transmission electron microscopy
EPR: Enhanced permeability and retention
GNP: Gold nanoparticle
